# An Abnormally Long Styloid Process Without Stylohyoid Ligament Ossification: Morphological and CT Findings from Two Dry Skulls

**DOI:** 10.3390/life15121795

**Published:** 2025-11-24

**Authors:** Andrei Ionut Cucu, Catalin Mihai Buzduga, Alexandru Carauleanu, Sorin Axinte, Alexandru Nemtoi, Gina Madalina Toma, Roxana Covali, Amelian Mădălin Bobu, Anca Sava, Iulian Prutianu, Camelia Tamas, Claudia Florida Costea, Angela Simalcsik

**Affiliations:** 1 Faculty of Medicine and Biological Sciences, Stefan Cel Mare University of Suceava, 720229 Suceava, Romania; andrei.cucu@usm.ro (A.I.C.); axintesorin@gmail.com (S.A.); alexandru.nemtoi@usm.ro (A.N.); toma.gina24@gmail.com (G.M.T.); 2 Faculty of Medicine, Grigore T. Popa University of Medicine and Pharmacy Iasi, 700115 Iasi, Romania; ale.carauleanu@umfiasi.ro (A.C.); amelian.bobu@gmail.com (A.M.B.); sava.anca@umfiasi.ro (A.S.); pruty04@gmail.com (I.P.); camelia.tamas@umfiasi.ro (C.T.); claudia.costea@umfiasi.ro (C.F.C.); 3 Clinical Emergency Hospital St. Spiridon, 700111 Iasi, Romania; 4 Suceava County Emergency Clinical Hospital, 720237 Suceava, Romania; 5 Faculty of Medical Bioengineering, Grigore T. Popa University of Medicine and Pharmacy Iasi, 700115 Iasi, Romania; ana.covali@umfiasi.ro; 6 Olga Necrasov Centre of Anthropological and Biomedical Research, Romanian Academy Iași Branch, 700479 Iasi, Romania; angellisimal@gmail.com; 7 Orheiul Vechi Cultural-Natural Reserve, MD 3552 Butuceni, Moldova; 8 Ion Niculiță Center of Archaeology, State University of Moldova, MD 2009 Chisinau, Moldova; 9 Institute of Bioarchaeological and Ethnocultural Research, MD 2008 Chisinau, Moldova

**Keywords:** elongated styloid process, Reichert’s cartilage, tympanohyal segment, Eagle’s syndrome, stylohyoid complex

## Abstract

Background: The styloid process is a slender, cylindrical bony projection of the temporal bone, showing marked interindividual variability in length, orientation, and degree of ossification. Its abnormal elongation, defined as exceeding 30 mm, is often associated with Eagle’s syndrome but may also occur as an incidental anatomical variant. Objective: This study reports two rare cases of abnormally long styloid processes without ossification of the stylohyoid ligament, identified in adult dry skulls from the osteological collection of the “Olga Necrasov” Centre of Anthropological Research, Iași, Romania, and provides morphological and CT-based characterization. Materials and Methods: Both skulls were examined macroscopically and by CT, with 3D reconstructions being used for morphometric analysis. Results: In Case 1, the left SP measured 62 mm, corresponding to Langlais type I elongation, with no evidence of pseudoarticulation or ligamentous ossification. In Case 2, the left SP was elongated to 33 mm and fusiform in shape, while the contralateral (right) SP was completely absent, a highly uncommon anatomical variation scarcely reported in the literature. Both findings were confirmed by CT imaging. Conclusions: The coexistence shows that the stylohyoid complex cand vary greatly during development. Such findings expand the spectrum of known anatomical variants of the stylohyoid complex and underscore the importance of detailed morphologic and imaging evaluation of the styloid region in both anatomical and clinical contexts.

## 1. Introduction

The styloid process (SP) is a cylindrical bony projection that originates from the inferior aspect of the petrous part of the temporal bone, located anterior to the stylomastoid foramen [1]. This process holds significant morphological and clinical importance, as its anatomical variations in length, orientation, and degree of ossification may give rise to various clinical manifestations collectively known as Eagle’s syndrome.

The normal length of the SP typically ranges between 20 and 25 mm, while processes exceeding 30 mm are considered elongated. The study of these anatomical variants, particularly in osteological material, provides valuable insights for anatomy, paleoanthropology, medical imaging, and cervicofacial surgery.

### 1.1. Anatomy and Embryology

**Figure 1:**
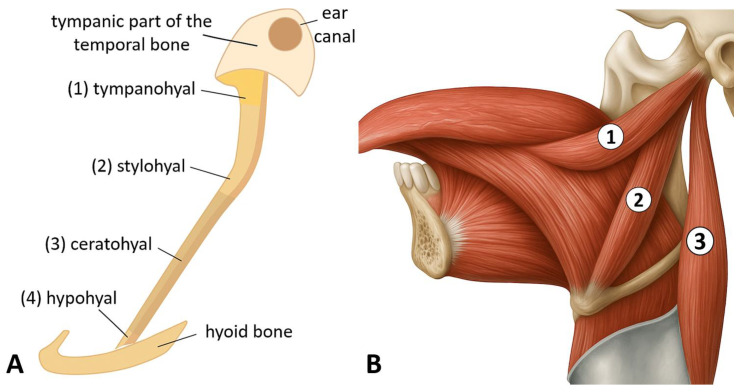
(**A**) Components of the stylohyoid chain derived from Reichert’s cartilage: *tympanohyal*, *stylohyal*, *ceratohyal*, and *hypohyal* segments. (**B**) Riolan’s styloid bundle, showing the *styloglossus* (1), *stylohyoid* (2), and *stylopharyngeus* (3) muscles (*images by Andrei Cucu*).

From an embryological standpoint, the SP develops from Reichert’s cartilage of the second pharyngeal arch [2]. This portion of Reichert’s cartilage consists of four developmental centers: (1) the tympanohyal, (2) the stylohyal, (3) the ceratohyal, and (4) the hypohyal, which give rise to the distinct components of the stylohyoid chain. Specifically, the tympanohyal forms the base of the SP, the stylohyal develops into its shaft, the ceratohyal gives rise to the stylohyoid ligament, and the hypohyal forms the lesser horn of the hyoid bone [3] (Figure 1A).

Anatomically, the SP is located in the lateral region of the neck, positioned between the internal and external carotid arteries and in close proximity to the internal jugular vein. Several cranial nerves, including the facial, glossopharyngeal, vagus, and hypoglossal nerves are situated near the SP [4]. The distal portion of the process serves as the origin of several muscles, including the styloglossus, stylohyoid, and stylopharyngeus, which play essential roles in deglutition and phonation [5] (Figure 1B).

In addition, two ligaments originate from the SP, the stylomandibular and stylohyoid ligaments, which insert on the ramus of the mandible and the lesser horn of the hyoid bone, respectively [6]. These muscles and ligaments attached to the SP are collectively known as “Riolan’s bouquet”, first described by the anatomist Jean Riolan the Younger (1577–1657) in the first half of the 17th century [7]. Within this complex, the three muscles, styloglossus, stylohyoid, and stylopharyngeus, are referred to as the “red flowers”, whereas the two ligaments, the stylomandibular and stylohyoid, are termed the “white flowers” [7,8]. This musculoligamentous complex plays an essential role in the movements of the oropharyngeal apparatus. The styloglossus muscle contributes to the retraction and lateral elevation of the tongue, the stylohyoid muscle elevates the hyoid bone during swallowing, and the stylopharyngeus muscle elevates and widens the pharynx during deglutition, also assisting in laryngeal elevation [6]. Conversely, the stylomandibular ligament limits excessive mandibular opening and protrusion, while the stylohyoid ligament connects the SP to the hyoid bone and assists in its elevation [9].

### 1.2. Mechanisms of Styloid Process Elongation

The elongation of the SP is a complex phenomenon involving multiple anatomical and molecular mechanisms, influenced by genetic and environmental factors as well as by mechanical stresses during development. Morphological studies have shown that elongated styloid processes (ESPs) often contain calcified cartilaginous islands and fibrous tissue, suggesting a congenital basis for elongation, possibly influenced by mechanical forces acting during fetal development [10,11].

Ghosh and Dubey analyzed the mechanism underlying SP elongation [12] and found that it originates from the cartilage of the second branchial arch, which consists of four segments with notable variations: tympanohyal, stylohyal, ceratohyal, and hypohyal. When both the tympanohyal and stylohyal segments undergo ossification within the first eight years of life, a longer SP results. Conversely, if only the tympanohyal segment ossifies while the stylohyal and ceratohyal portions resorb, the process remains shorter [12]. Although it was long believed that SP elongation might be due to postnatal growth, subsequent research has not confirmed a clear correlation between its length and age [13].

Immunohistochemical studies have identified an increased expression of osteogenic proteins such as osteonectin, osteocalcin, BMP-2, BMP-4, and RANKL in ESPs. These proteins are associated with bone growth and remodeling, indicating a molecular basis for the elongation phenomenon [14]. Among them, bone morphogenetic proteins (BMPs), particularly BMP-2 and BMP-4, play a critical role in regulating osteogenesis and are strongly expressed in ESPs. These proteins induce osteoblastic differentiation and are involved in signaling pathways that promote bone formation [15,16]. Similarly, osteocalcin and osteonectin serve as markers of bone formation and are found at high levels in the periosteal membrane of ESPs, reflecting active bone remodeling [15]. The protein RANKL contributes to bone resorption and remodeling, suggesting a dynamic balance between bone formation and resorption during the elongation process. Moreover, the presence of protective stress-related proteins such as HO-1, HSP-70, and HSP-90 indicates a cellular response to increased tractional stress in the ligaments attached to the SP, which may further trigger or enhance elongation [15].

### 1.3. Epidemiological Variability of the Elongated Styloid Process

The SP has an average length ranging between 20 and 25 mm [17]. Its elongation, referred to as the elongated styloid process (ESP), represents an anatomical variant in which the SP exceeds 30 mm in length when measured from its point of emergence on the temporal bone to the tip of the process. This threshold of 30 mm is widely accepted across most studies as the defining criterion for elongation [18,19,20].

The ESP was first described in 1652 by the Italian surgeon Pietro Marchetti (1589–1673), who identified it as an ossification of the stylohyoid ligament [17,21]. Marchetti also provided the first clinical description associated with an ESP, reporting cases of intermittent respiratory distress linked to this anatomical variation [22].

Regarding the prevalence of ESP, numerous anatomical and radiological studies across various populations have reported rates ranging from 3.3% to 84.4% [19,20,23,24,25,26,27,28,29,30,31,32,33,34,35,36,37,38,39,40,41,42,43,44,45,46,47,48,49,50,51,52,53,54]. This wide range in prevalence is largely attributed to methodological and technical differences, including variations in imaging modalities, sample selection, and the use of surgical specimens or osteological materials (cadaveric or dry skulls) [24,29].

The ESP demonstrates marked geographical variability, with prevalence differing not only between continents but also among regions within the same country. A study conducted in Spain reported a high prevalence of 72.75% among the population of Barcelona, based on digital panoramic radiographs [55]. In Eastern Europe, Kapur et al. reported a significantly lower prevalence of 7% in a Bosnian population, based on an analysis of 200 dry skulls, suggesting regional variability even within Europe [56]. In South America, several studies have revealed interregional differences within Brazil. In the Bahia region, the prevalence of ESP was 29.6% [57], whereas in Paraíba, it reached 32% [58]. High prevalence rates have also been documented in Colombia (50%) [59] and in the Aseer region of Saudi Arabia (63.2%) [40]. Interestingly, a separate study from the Riyadh region of Saudi Arabia reported a lower prevalence of 27.3% [60]. In India, one study found a prevalence of 32.53% [61], while in Turkey, a lower rate of 16.3% was observed [62].

In Romania, a single study by Andrei et al. evaluated 88 styloid processes (SPs) from 44 patients using 3D cone-beam computed tomography and reported that approximately 40% of the processes measured over 35 mm in length [63]. These findings collectively suggest that the prevalence of ESP varies substantially not only across continents but also among geographic regions within the same country, being influenced by age, sex, genetic background, and environmental factors.

Age-related differences have also been documented, with several studies indicating a higher prevalence of ESP among older adults. Santana et al. reported a prevalence of 40.6% in older adults compared with 26.3% in younger individuals within a Brazilian cohort [57]. Other studies across different populations similarly demonstrated an age-associated increase in ESP prevalence [18,44,62]. Some authors also noted a significant positive correlation between SP length and age [62].

Regarding sex-related differences, findings remain inconsistent across studies. Some authors have reported a higher prevalence in males [58,64], while others observed a greater prevalence in females, particularly within the 51–70-year age group [65]. However, recent meta-analyses have found no statistically significant sex predilection [66].

In terms of laterality, results are equally variable. In a study involving 498 patients, a strong linear correlation was found between the lengths of the right and left SPs, with a slightly higher prevalence on the right side (37.34% in males and 29.18% in females) [67]. Another study on 50 dry skulls reported that the right SP tended to be longer than the left, although the difference was not statistically significant [68]. Conversely, a study of a Jamaican population found that the mean length of ESPs was greater on the left side (4.59 cm) compared with the right (3.58 cm), suggesting possible population-specific variations [64].

The present study describes two rare cases of abnormally ESPs without ossification of the stylohyoid ligament, identified in adult dry skulls from the Osteological Collection of the “Olga Necrasov” Center for Anthropological and Biomedical Research (Iași, Romania), belonging to the Romanian Academy. The primary aim of this study was to provide a morphological and radiological (CT-based) characterization of these anatomical variants.

## 2. Materials and Methods

This study was conducted on two osteological collections housed in the osteological collection of the “Olga Necrasov” Center for Anthropological and Biomedical Research in Iași (Romania).

The first collection originates from the medieval-modern necropolis of Roman-Arhiepiscopie (Neamț County, Romania), excavated in 2015 and dated archeologically to the 15th–17th centuries. A total of 226 human skeletons were analyzed (57 adult males, 63 adult females, and 106 non-adults), recovered from 210 inhumation graves [69,70].

The second osteological collection originates from the medieval-modern necropolis of Cîrligi (Neamț County, Romania), dated to the 17th–18th centuries. In 2020, seven inhumation graves were excavated, yielding the skeletal remains of seven individuals (one adult female, two adult males, and four non-adults) [71].

All skulls were macroscopically examined to identify anatomical and congenital variations in the SPs of the temporal bones. Among these specimens, two skulls were found to exhibit multiple anatomical variants of the SP, as well as other osseous anomalies at the cranial level. Photographs were taken to document these morphological variants, and both skulls underwent computed tomography (CT) scanning for detailed imaging analysis. The length of the SP, defined as the distance between its base and tip, was measured using both a digital caliper and cranial CT images. The inferior margin of the tympanic bone was used as the reference point for all measurements. A comprehensive summary of the CT acquisition parameters, reconstruction settings, and measurement protocol applied in this study is presented in Table 1.

## 3. Results

From the total of 233 (226 + 7) dry skulls, the following anatomical variations in the SP of the temporal bone were observed.

### 3.1. Case No. 1

**Figure 2:**
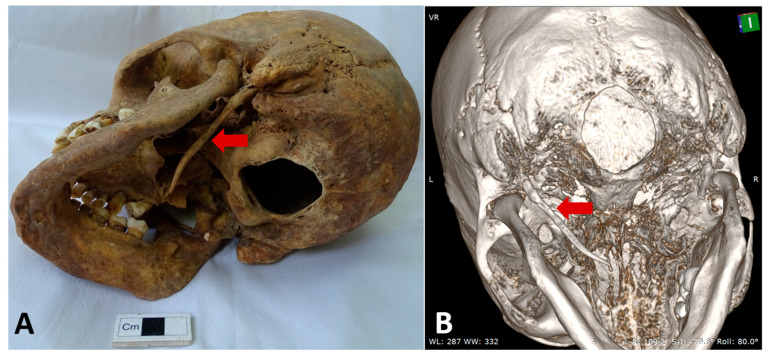
Case No. 1. (**A**) Inferolateral left view of the skull showing the elongated left styloid process (red arrow). (**B**) Left oblique view of the 3D reconstruction highlighting the elongated left styloid process (red arrow).

Case 1 was observed in male individual, with an estimated age at death of 50–60 years (maturus III). Examination revealed an elongated, uninterrupted SP on the left side, without signs of pseudoarticulation or segmentation, measuring 62 mm in length, consistent with a Langlais type I ESP (Figure 2A). The right SP was found to be fractured. Associated osseous pathologies included generalized degenerative osteoarthritis, most pronounced in the spine, with advanced degenerative changes in the lumbar vertebrae. No medical history or clinical background data were available for this individual. The case was identified in the flat medieval-modern inhumation necropolis from the locality of Roman, site Arhiepiscopie (Neamț County, Romania). Cranial CT examination and 3D reconstructions of skull No. 1 confirmed the elongation of the left SP (Figure 2B).

**Figure 3:**
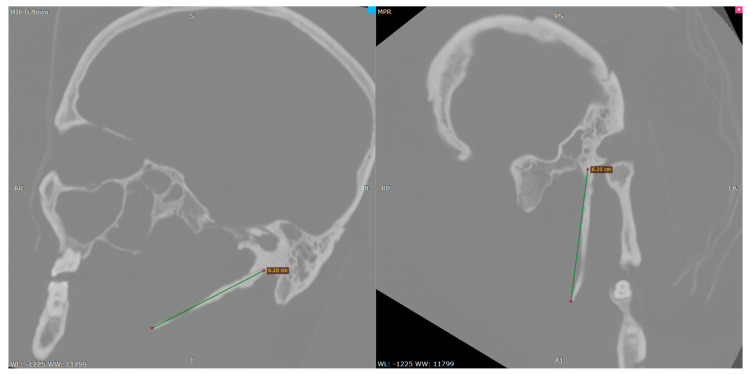
Sagittal (**left**) and oblique (**right**) multiplanar CT reconstructions demonstrating the linear measurement of the left styloid process.

For the left SP, measurements were obtained using multiplanar reconstructions (MPR) aligned to the orbito-meatal sagittal plane, with additional oblique adjustments in the axial plane to accurately follow the anatomical curvature of the process. Linear length was measured from the inferior surface of the petrous part of the temporal bone to the distal tip of the SP The anatomical landmarks, reference planes, and 3D reconstruction values used for assessing the left SP are summarized in Table 2 and illustrated in Figure 3.

### 3.2. Case No. 2

**Figure 4:**
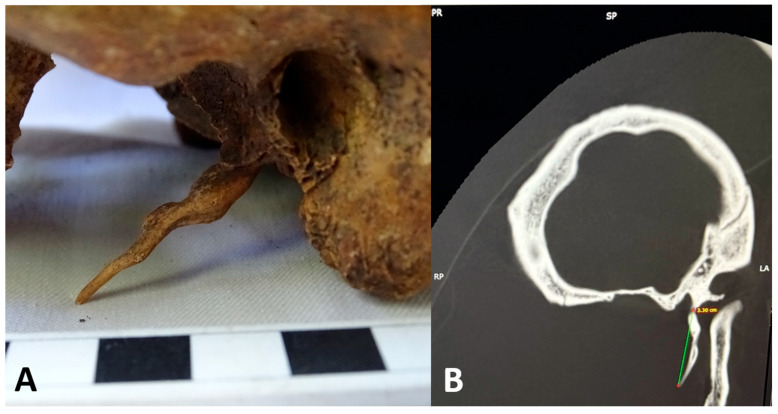
Case No. 2. (**A**) Left lateral close-up view highlighting the elongated left styloid process (detailed view). (**B**) Multiplanar CT reconstructions showing linear measurement of the left styloid process.

Case 2 was observed in male individual, with an estimated age at death of 45–50 years (maturus II–III). The skeleton originates from the medieval-modern necropolis of Cîrligi (Neamț County, Romania). Examination revealed an elongated, curved, and fusiform-shaped left SP, measuring 33 mm in length (Figure 4).

The specific landmarks, reference planes, and 3D reconstruction values applied in this assessment are summarized in Table 3.

**Figure 5:**
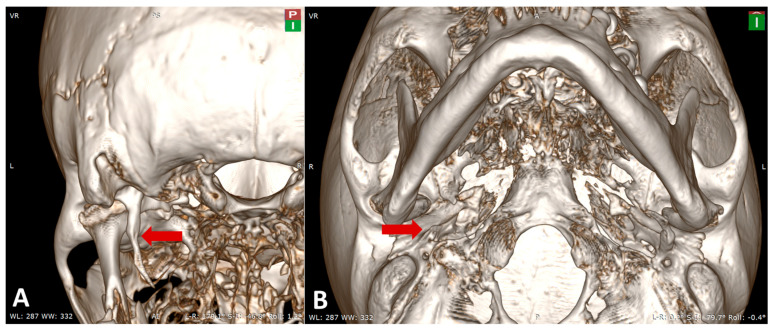
Case No. 2. (**A**) Posterior view of the 3D-reconstructed skull showing the elongated left styloid process (red arrow). (**B**) Inferior view of the 3D reconstruction demonstrating the absence of the right styloid process (red arrow).

Subsequent cranial CT examination (bone window), confirmed a type I ESP according to Langlais’ classification, as trabecular continuity was observed along the length of the SP. Also, the CT examination of the skull confirmed the absence of the right SP (Figure 5).

**Figure 6:**
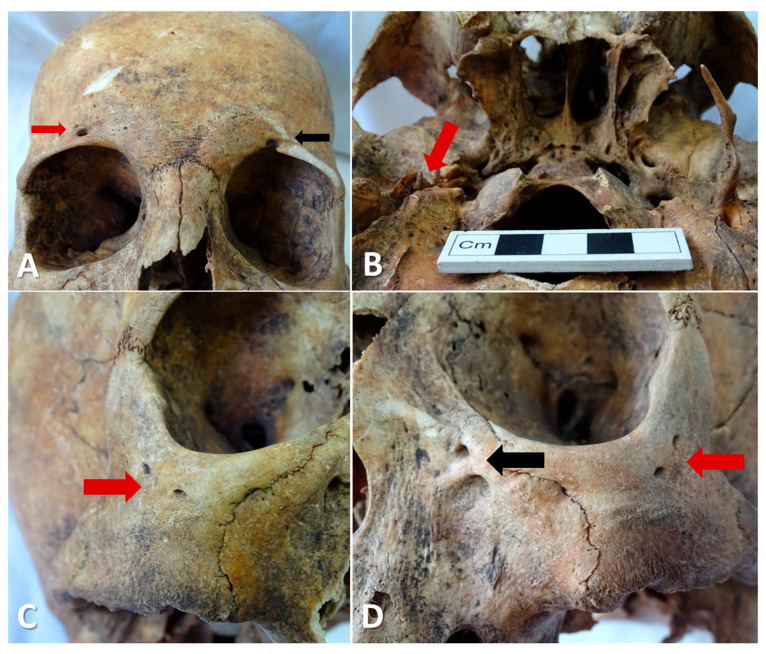
Case No. 2. (**A**) Anterior view of the skull showing the enlarged right supraorbital foramen (red arrow) and the left supraorbital sulcus (black arrow). (**B**) Basal view of the skull displaying the elongated left styloid process and the absence of the contralateral styloid process (red arrow). (**C**) Right oblique anterolateral view showing a double zygomatic foramen (red arrow). (**D**) Left oblique anterolateral view showing a double zygomatic foramen (red arrow) and a double left infraorbital foramen (black arrow).

Examination of skull No. 2 revealed several cranial anatomical variants, the most notable being: a widened right supraorbital foramen, a left supraorbital sulcus, bilateral double zygomatic foramina, and a double left infraorbital foramen (Figure 6).

To provide quantitative context and to address the variability of these findings within the broader sample, Table 4 summarizes the morphometric characteristics of the two cases in comparison with all 233 skulls analyzed in the osteological collection.

## 4. Discussion

### 4.1. Type and Calcification Patterns of Elongated Styloid Processes

From a structural perspective, ESPs can be classified into two main types: type I—a truly elongated SP, and type II—an ossified stylohyoid ligament. The type I ESP, in which the SP itself is elongated, is characterized by bony hyperplasia and a well-defined trabecular architecture [72]. Type I elongation is considered the most common form of SP elongation in various studies, with reported prevalence rates ranging from 65.85% to 73.1% [73,74]. It occurs more frequently in females than in males [20,49], and is most often bilateral, involving elongation of both SPs [74,75]. From a clinical standpoint, many type I ESPs are asymptomatic, but some individuals may experience head and neck pain. This pain may be misinterpreted as being of dental or otorhinolaryngological origin [37,76].

The type II ESP, also known as the ossified stylohyoid ligament, results from metaplastic transformation of the stylohyoid ligament, leading to the formation of bone-like tissue that lacks the typical compact structure found in normal bone [72]. The ultrastructure of type II ESP shows no trabecular architecture, indicating a distinct ossification mechanism compared to type I, which involves osseous hyperplasia. Micro-CT studies have demonstrated that type II ESPs exhibit a lower bone-to-soft tissue ratio than type I, a feature that facilitates differential diagnosis between the two types Clinically, type II ESPs may generate cervicofacial pain, dysphagia, and other symptoms related to neurovascular compression [72].

**Figure 7:**
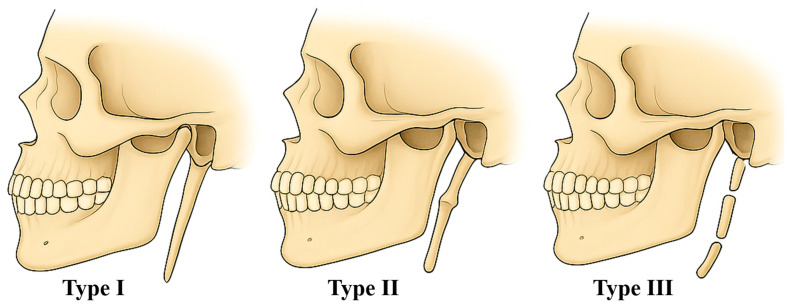
Morphological classification of type of styloid process by Langlais et al. (1986) [77]. Type I—uninterrupted, type II—pseudoarticulated, type III—segmented (*images by Andrei Cucu*).

Regarding the morphology of ossification, Langlais et al. (1986) proposed a three-type classification of the SP based on its radiological appearance [77]. Type I (elongated): refers to a continuous, uninterrupted elongation of the SP without segmentation. It represents the simplest and most common form of SP elongation and can be easily identified on radiographic images. Type II (pseudoarticulated): is characterized by an apparent joint (pseudoarticulation) within the SP, giving it a segmented appearance that may sometimes be mistaken for a fracture. Type III (segmented): consists of distinct, separate segments, which may result from partial ossification or developmental anomalies [77] (Figure 7).

The length of the SP is an anatomically significant feature due to its potential clinical implications, particularly in relation to Eagle’s syndrome. Although the average length of the SP is approximately 25 mm, with variations depending on the studied population [47,56,59,78], the literature reports rare cases in which the SP significantly exceeds this value, sometimes being associated with ossification of the stylohyoid ligament. The table below illustrates the diversity of these morphological findings reported across different studies (Table 5).

In contrast to the cases summarized in Table 5, the present specimens combine several rare features. First, the left SP in Case 1 measures 62 mm and is one of the longest type I ESPs reported in dry skull material without any stylohyoid ligament ossification. Second, Case 2 combines a left-sided type I ESP (33 mm) with complete aplasia of the contralateral process. This laterality pattern has been described only sporadically in the literature and remains exceptional. Taken together, our two cases lie at the extreme upper limit of the currently known morphometric spectrum of the stylohyoid complex and show that elongation and absence can coexist in the same individual.

From a developmental perspective, our observations highlight the considerable variability of the bony segments derived from Reichert’s cartilage. In Case 1, the markedly elongated SP (62 mm) without stylohyoid ligament ossification suggests accentuated development of the tympanohyal and stylohyal segments, which normally form the base and shaft of the process. This exaggerated growth likely reflects an individual variation in the degree of early postnatal maturation and primary ossification of these segments. In Case 2, the complete absence of the right SP points to an early disturbance in the development of the tympanohyal segment. The unusual combination of unilateral elongation with contralateral aplasia demonstrates that derivatives of the second branchial arch may show extreme asymmetry within the same individual. At the population level, analysis of the entire osteological collection (n = 233 skulls) revealed a very low prevalence of such variants: ESPs (>30 mm) were found in only 2 skulls (0.86%), and complete absence of the SP was observed in a single skull (0.43). These frequencies are much lower than the prevalence values reported in modern radiological studies, where elongation commonly ranges between 3% and 30%.

In addition to these developmental aspects, a biomechanical contribution may also help explain the marked asymmetry observed in Case 2. Asymmetric loading of the styloglossus, stylohyoid, and stylopharyngeus muscles, as well as subtle differences in mandibular and hyoid motion during growth, could locally modulate periosteal activity and bone modeling. Such factors might favor elongation on one side while the contralateral SP fails to develop or remains rudimentary. Although this hypothesis cannot be tested directly in archeological material, it offers a plausible developmental-biomechanical framework for understanding how elongation and aplasia can occur together in the same skull.

While the elongation of the SP is primarily driven by osteogenic proteins and their associated molecular signaling pathways, it is essential to consider the broader context of bone growth and remodeling. Mechanical stress, hormonal influences, and genetic predisposition are likely to act together with molecular factors rather than in isolation. A better understanding of the interaction between these mechanisms may provide useful insights for future therapeutic strategies in conditions such as Eagle’s syndrome. Anatomical variations in the length of the SP hold notable anatomical, anthropological, and clinical importance. Such anomalies may compress or irritate adjacent neurovascular structures, leading to a range of symptoms including cervical pain, dysphagia, odynophagia, otalgia, facial pain, headache, tinnitus, or trismus, a constellation of manifestations first described in 1937 by Watt W. Eagle as a distinct clinical entity, now known as Eagle’s syndrome [82].

Eagle classified this syndrome into two subtypes: the classic type and the carotid type [83,84,85,86]. In the classic type, the most common symptoms include dysphagia, odynophagia, and a foreign-body sensation in the throat [22,87]. In the carotid type, compression of the internal carotid artery may cause pain in the parietal region of the skull or in the periorbital area [88]. Both subtypes are associated with the ESP but differ in their clinical manifestations and underlying mechanisms. The classic variant is mainly characterized by pain-related symptoms such as odynophagia, neck pain, and otalgia, resulting from cranial nerve irritation or compression. The carotid variant, by contrast, involves vascular complications due to compression or irritation of the carotid artery, and may lead to neurological manifestations, including transient ischemic attacks [89,90]. According to several authors, the ESP becomes symptomatic when the SP exceeds 40 mm in length [27,91], and among all individuals with an elongated SP, approximately 4–10.3% may develop painful or symptomatic forms of Eagle’s syndrome [92].

Even though our observations are based on dry skulls without clinical records, the extreme morphologies documented in Cases 1 and 2 correspond to configurations that, in living patients, would likely be considered at increased risk for Eagle’s syndrome or for iatrogenic complications during surgical or endoscopic procedures in the parapharyngeal space. This underlines the relevance of recognizing such variants in radiological and anatomical practice.

### 4.2. Anatomical Variants Associated with Elongated Styloid Process

The ESP is an anatomical variant that may occur in association with other osseous anomalies, particularly involving the stylohyoid ligament and the hyoid bone. Most commonly, SP elongation is accompanied by calcification of the stylohyoid ligament, which may be partial, complete, or nodular, thereby altering the morphology of the SP [93]. Additionally, the SP may form part of the hyoid apparatus, involving divisions such as the stylohyal and ceratohyal segments. These associations can lead to variations in both the length and morphology of the SP [91].

Examination of skull no. 2, which presented a left ESP, also revealed the absence of the contralateral (right) SP. Regarding the absence of the SP, Ramadan et al. reported, in a sample of 100 patients (analyzing 200 SPs), two cases of bilateral absence and one case of unilateral absence of the SP [94]. Although these two anatomical variations, elongation and absence of the SP—have been predominantly documented independently, a few studies have described rare cases in which one SP was elongated while the contralateral one was absent [95,96]. The authors emphasized that such complete absence of the SP may result from a developmental anomaly of the tympanohyal segment, from which the process normally derives [96]. This particular combination, an ESP on one side and absence of the contralateral process, expands the known spectrum of anatomical variations within the stylohyoid complex and underscores the importance of detailed morphological studies of the cranial base.

In the same skull no. 2, several additional osseous anatomical variants were observed, the most notable being: a widened right supraorbital foramen, a left supraorbital sulcus, bilateral double zygomatic foramina, and a double left infraorbital foramen. Although the coexistence of these features might suggest a shared morphogenetic relationship, embryological evidence does not support a direct correlation. The SP derives from the second branchial arch, whereas the foraminal variants originate from structures developed from the first branchial arch. Therefore, the coexistence of these morphological forms most likely reflects an individual predisposition to skeletal variability, falling within the normal morphological polymorphism of the human skull.

Taken together, the association of an elongated left SP, contralateral aplasia, and multiple cranial foraminal variants in Case 2 suggests a generalized tendency toward skeletal variability rather than a single localized defect. By documenting these combined anatomical findings with detailed CT-based morphometry, our study adds a new, well-characterized example to the small number of reports describing such complex variants of the stylohyoid complex and cranial base.

## 5. Conclusions

The present study contributes new data on the morphological variability of the SP through the description of two rare cases identified in osteological material from the “Olga Necrasov” Center for Anthropological and Biomedical Research Collection in Iași, Romania. In both cases, the SP exhibited type I characteristics according to the Langlais classification, without ossification of the stylohyoid ligament, findings that were confirmed by cranial CT examination and 3D reconstruction.

The first case revealed a left SP measuring 62 mm, placing it among the longest processes reported in the international literature, whereas the second case presented a 33 mm left ESP associated with complete absence of the contralateral right process, an exceptional anatomical variation that has been only sporadically described previously.

The identification of such anatomical variations in osteological material has significant implications for anatomy, paleoanthropology, and medical imaging, emphasizing the importance of thorough evaluation of the styloid region in clinical practice. In particular, radiologists, maxillofacial surgeons, otorhinolaryngologists, and neurosurgeons should be aware that extreme elongation or agenesis of the styloid process may alter the expected anatomy of the skull base and parapharyngeal space, influence the interpretation of CT and CBCT studies, and contribute to cervicofacial pain syndromes compatible with Eagle’s syndrome. Recognition of these variants may therefore help to avoid misdiagnosis, improve preoperative planning, and reduce the risk of intraoperative complications.

Future research, based on larger samples and advanced morphometric methods, may provide deeper insights into the developmental mechanisms and clinical significance of these rare anomalies and clarify which specific morphometric thresholds are most strongly associated with symptomatic presentations.

## Figures and Tables

**Table 1:** CT acquisition and measurement parameters.

Parameter	Description
Acquisition type	Spiral
Slice thickness	Total: 1.25 mm; Single collimation: 0.62 mm
kVp	140 kV
Resolution	—
Pitch	0.56
Software	Reconstruction performed in RADIANT (version 2025.2)
Scanner	GE Medical Systems
Dose	18.22 Gy (Case No. 1) and 117.76 mGy (Case No. 2)

**Table 2:** CT-based measurement landmarks and reference planes for the left styloid process (Case No. 1).

Parameter	Description
Anatomical landmarks	Multiplanar reconstruction using the orbito-meatal sagittal plane, with oblique adjustment of the axial plane. Length: 62 mm measured in the sagittal plane; the distal portion shows a slight curvature.
Reference planes	Dimensional assessment from the inferior surface of the petrous part of the temporal bone
3D Model	3D reconstruction—length: 61 mm

**Table 3:** CT-based measurement landmarks and reference planes for the left styloid process (Case No. 2).

Parameter	Description
Anatomical landmarks	MPR reconstruction using the orbito-meatal sagittal plane, with oblique adjustment in the axial plane. Length: 33 mm measured in the coronal plane.
Reference planes	Dimensional assessment from the inferior surface of the petrous part of the temporal bone
3D Model	3D reconstruction—length: 31.2 mm

**Table 4:** Comparative overview of styloid process morphology in the two reported cases versus the full osteological sample (n = 233 skulls).

Parameter	Case 1	Case 2—Left SP *	Case 2—Right SP *	Entire Collection (n = 233)
Presence of styloid process	Present bilaterally (right fractured)	Present	Absent	Present in 232 skulls (99.57%); absent in 1 skull (0.43%)
Length (mm)	62 mm	33 mm	—	Only 2 skulls with SP > 30 mm (0.86% of sample)
Type (Langlais classification)	Type I	Type I	—	Majority Type I; no elongated SPs except Cases 1 and 2
Shape	Straight, non-segmented	Curved, fusiform	—	Mostly normal (20–25 mm), no fusiform elongations observed
Stylohyoid ligament ossification	Absent	Absent	—	Not observed in any other skull
Laterality pattern	Unilateral elongation	Unilateral elongation	Contralateral aplasia	No other skulls with this combination

* SP—styloid process.

**Table 5:** Reported cases of elongated styloid processes (>50 mm).

Study, Year	Location	Elongated Styloid Process	Length (mm)	Stylohyoid Ligament Ossification	Sex/Age	Type of Specimen
Current study (2025)	Unilateral	Left	62	No	N/A	Dry skull
Guarna and Aglianò (2018) [3]	Bilateral	Right Left	50 70	Yes	N/A	Dry skull
Kusunoki et al. (2016) [13]	Bilateral	Right Left	78 80	No	M/68	CT-scanned skull
Bagoji et al. (2013) [10]	Bilateral	Right Left	60 59	N/A	N/A	Dry skull
Hosokawa et al. (2011) [79]	Unilateral	N/A	70	Yes	F/68	CT-scanned skull
Kolagi et al. (2010) [80]	Bilateral	Right Left	80 N/A	Yes	N/A	Dry skull
	Unilateral	Left	63	N/A	M	Fresh skull
Paraskevas et al. (2009) [14]	Unilateral	Right	58	Yes	M/72	Dry skull
Prabhu et al. (2007) [81]	Bilateral	Right Left	60 59	No No	F/adult	Dry skull
Shamir et al. (2009) [64]	(specimen 1) Bilateral	Right Left	45 63	No No	M/60	Dry skull

## Data Availability

The original contributions presented in this study are included in the article. Further inquiries can be directed to the corresponding author.
